# Heparin-induced thrombocytopenia in extracorporeal membrane oxygenation-supported patients: a systematic review and meta-analysis

**DOI:** 10.1186/s12959-024-00624-5

**Published:** 2024-06-28

**Authors:** Danyu Song, Yu Jin, Yang Zhang, Zhou Zhou

**Affiliations:** 1 Department of Laboratory Medicine, National Center for Cardiovascular Disease, Chinese Academy of Medical Sciences & Peking Union Medical College Fuwai Hospital, Fuwai Hospital, No. 167 Beilishi Road, Xicheng District, Beijing, 100037 China; 2 Department of Cardiopulmonary Bypass, National Center for Cardiovascular Disease, Chinese Academy of Medical Sciences & Peking Union Medical College Fuwai Hospital, Beijing, China

**Keywords:** Extracorporeal membrane oxygenation (ECMO), Heparin-induced thrombocytopenia (HIT), Thrombocytopenia

## Abstract

**Background:**

In recent years, extracorporeal membrane oxygenation (ECMO) has been increasingly used in critically ill patients with respiratory or cardiac failure. Heparin is usually used as anticoagulation therapy during ECMO support. However, heparin-induced thrombocytopenia (HIT) in ECMO-supported patients, which results in considerable morbidity and mortality, has not yet been well described. This meta-analysis and systematic review aimed to thoroughly report the incidence of HIT on ECMO, as well as the characteristics and outcomes of HIT patients.

**Methods:**

We searched the PubMed, Embase, Cochrane Library, and Scopus databases for studies investigating HIT in adult patients supported by ECMO. All studies conforming to the inclusion criteria were screened from 1975 to August 2023. Nineteen studies from a total of 1,625 abstracts were selected. The primary outcomes were the incidence of HIT and suspected HIT.

**Results:**

The pooled incidence of HIT in ECMO-supported patients was 4.2% (95% CI: 2.7–5.6; 18 studies). A total of 15.9% (95% CI: 9.0-22.8; 12 studies) of patients on ECMO were suspected of having HIT. Enzyme-linked immunosorbent assay (ELISA) is the most commonly used immunoassay. The median optical density (OD) of the ELISA in HIT-confirmed patients ranged from 1.08 to 2.10. In most studies, the serotonin release assay (SRA) was performed as a HIT-confirming test. According to the subgroup analysis, the pooled incidence of HIT in ECMO patients was 2.7% in studies whose diagnostic mode was functional assays, which is significantly lower than the incidence in studies in which the patients were diagnosed by immunoassay (14.5%). Argatroban was most commonly used as an alternative anticoagulation agent after the withdrawal of heparin. Among confirmed HIT patients, 45.5% (95% CI: 28.8–62.6) experienced thrombotic events, while 50.1% (95% CI: 24.9–75.4) experienced bleeding events. Overall, 46.6% (95% CI: 30.4–63.1) of patients on ECMO with HIT died.

**Conclusion:**

According to our study, the pooled incidence of HIT in ECMO-supported patients is 4.2%, and it contributes to adverse outcomes. Inappropriate diagnostic methods can easily lead to misdiagnosis of HIT. Further research and development of diagnostic algorithms and laboratory assays are warranted.

**Supplementary Information:**

The online version contains supplementary material available at 10.1186/s12959-024-00624-5.

## Introduction

Extracorporeal membrane oxygenation (ECMO) can provide short-term support for patients with severe respiratory or cardiac failure [1]. The contact between blood and nonendothelial surfaces during this process leads to the activation of coagulation. Circuit clotting and thromboembolic complications are frequently observed in ECMO-supported patients [2]. Therefore, anticoagulation therapy is necessary during ECMO support. According to the ELSO (Extracorporeal Life Support Organization) guidelines, unfractionated heparin (UFH) is recommended [3].

However, the use of heparin may lead to the development of heparin-induced thrombocytopenia (HIT). The symptom of HIT is moderate thrombocytopenia a few days after exposure to heparin, which paradoxically leads to an increased risk of thrombosis [4]. HIT is caused by antibodies attached to heparin–platelet factor 4 complexes [5]. HIT antibodies bind to platelet FcγRIIa [5], leading to platelet(PLT) activation and aggregation. However, during ECMO treatment, contact with foreign circuit surfaces and high shear stress also leads to the activation of PLT [4]. As previous studies reported, PLT counts decreased significantly after the initiation of ECMO and remained low throughout the entire process [6]. This makes clinical suspicion of HIT on ECMO challenging and may delay alternative treatment.

In adult patients treated with heparin, the incidence varies depending on the primary disease. Dhakal et al. reported that 0.065% of discharged patients were diagnosed with HIT [7]. According to the subgroup analysis, cardiopulmonary bypass (CPB) had the highest incidence, at 0.63%. The incidence of HIT in ECMO-supported patients is still not well described. HIT with ECMO support is challenging to diagnose, leading to adverse outcomes and missing data. The aim of this meta-analysis and systematic review was to probe the incidence of HIT on ECMO and the characteristics and outcomes of patients with HIT.

## Materials and methods

This systematic review and meta-analysis were conducted according to the Preferred Reporting Items for Systematic Reviews and Meta-Analysis (PRISMA) guidelines. Our study was registered on PROSPERO (CRD42022342374).

### Literature search and data extraction

The PubMed, Embase, Cochrane, and Scopus electronic databases were searched without language restrictions from 1975 to August 28th, 2023. The search strategy used the following terms: extracorporeal membrane oxygenation OR extracorporeal life support OR ECMO OR ECLS OR extracorporeal circulation OR extracorporeal AND heparin-induced thrombocytopenia OR HIT. Keywords and MeSH terms were used in relevant combinations.

The eligibility criteria for this systematic review and meta-analysis included the following: (1) Observational studies or randomized controlled trials with more than 10 adult patients. (2) The patients in the study were supported by ECMO due to various primary diseases, and heparin was used as anticoagulation therapy. (3) The study mentioned the situation of HIT on ECMO. (4) The diagnostic mode for HIT was clarified in the study. Reviews, animal studies, in vitro experiments, conference abstracts, and case reports were excluded. To avoid overlapping patient data, only the largest study was included.

Study selection was independently performed by two researchers (DS and YJ). If there was any disagreement, a third researcher (YZ) was involved in resolving the problem. We used a standard form to extract data from the included studies. Two researchers (DS and YJ) independently extracted the data. The extracted information included the author, year of publication, study type, institution, study period, sample size, patient characteristics, pre-ECMO clinical characteristics and laboratory parameters, incidence of HIT on ECMO, and characteristics and outcomes of patients with HIT.

### Outcomes

The primary outcome of this systematic review and meta-analysis was the incidence of HIT/suspected HIT on ECMO.

The secondary outcomes are diagnostic algorithms, immunoassays (methods and outcomes), functional assays, diagnostic modes of HIT, PLT counts of HIT patients, alternative anticoagulation and monitoring targets, the incidence of thrombotic and bleeding events in confirmed HIT patients, and mortality in confirmed HIT patients.

### Risk of bias

The Newcastle‒Ottawa Quality Assessment Scale, which was adapted for cross-sectional studies, was used to assess the quality of the included studies. The possibility of publication bias was assessed using Egger’s test, the visual assessment of funnel plots.

### Statistical analysis

STATA 16 was used to perform all meta-analyses of the incidence and outcomes of HIT. Because of the anticipated high degree of heterogeneity, we applied the DerSimonian–Laird random effects model. Continuous variables were described as means and standard deviation (SD) or medians and interquartile range (IQR). Pooled effect estimates (or odds ratio) were expressed as estimates (or odds ratio) with 95% confidence intervals (CIs). Heterogeneity was explored using the I^2^ statistic. We defined heterogeneity as follows: I^2^ = 0–50%, low heterogeneity; I^2^ = 50–75%, moderate heterogeneity; and I^2^ > 75%, high heterogeneity. We performed sensitivity analysis by omitting one study at a time to identify influential studies.

## Results

### Study selection

**Fig. 1:**
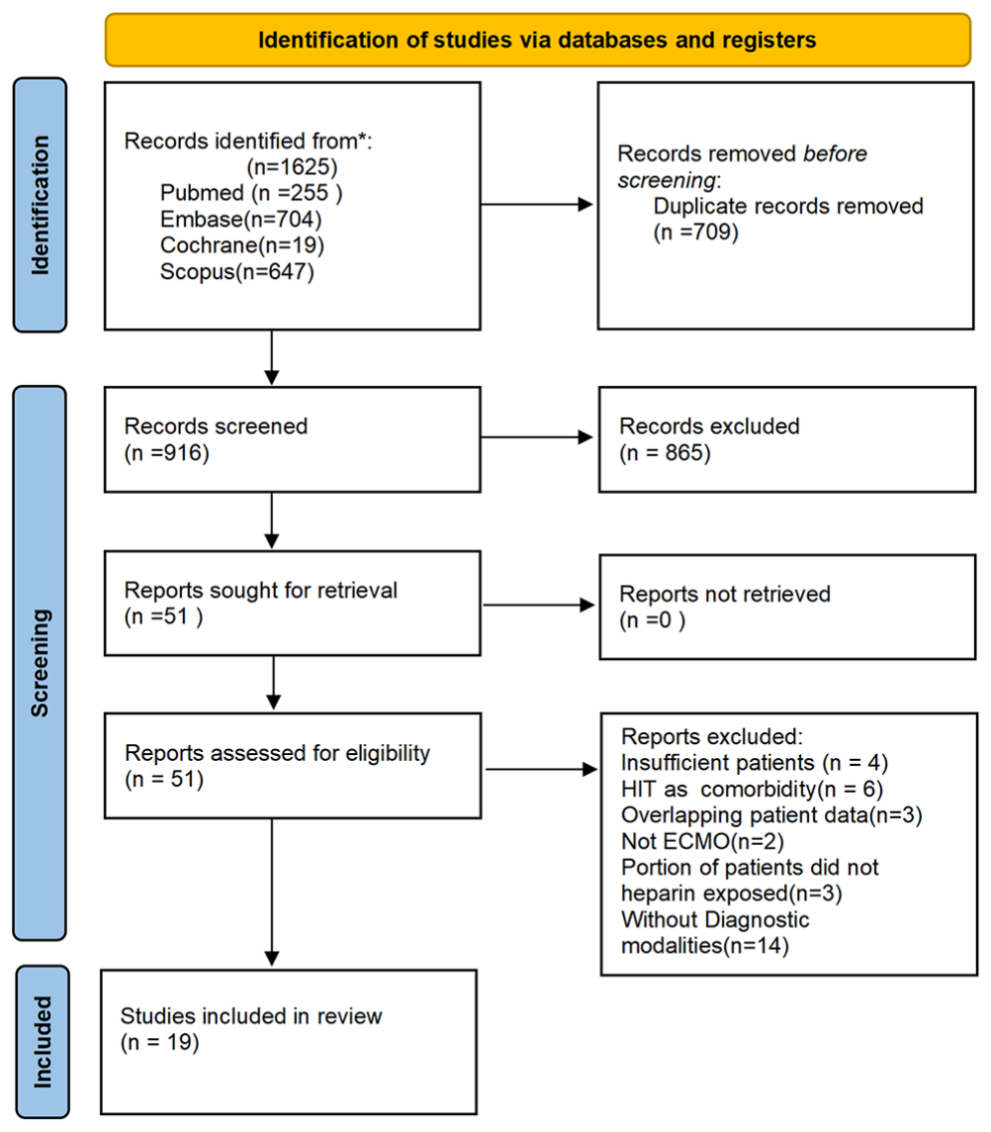
PRISMA schematic diagram of the search strategy

The process of study selection according to the inclusion and exclusion criteria is shown in the PRISMA flow diagram (Fig. 1). A total of 1,625 references were screened, and 51 studies were identified as potentially relevant studies whose full texts were retrieved. After removing studies that did not meet the inclusion criteria, 19 studies [8–26] with 9411 patients were included in the data assessment.

### Study description

The main characteristics of the included studies are shown in Supplementary Table 1. Table 1 shows the details of the outcomes. Although the authors did not report the ECMO type in 4 studies [14, 15, 19, 22], 1293 patients were supported by veno-venous extracorporeal membrane oxygenation (VV-ECMO), 6863 patients were supported by veno-arterial extracorporeal membrane oxygenation (VA-ECMO), and 2 patients were on other types of ECMO. Patient age ranged from 23 to 76 years. The average ECMO duration ranged from 4 to 17 days. Eighteen studies reported the incidence of HIT. Of these, 12 studies reported suspicions of HIT. All studies presented their diagnostic mode. All studies performed immunoassays, and 15 studies performed functional assays to confirm HIT. Diagnostic algorithms were described in 9 studies, and 11 studies presented the PLT count of HIT patients. Fifteen studies reported alternative anticoagulation therapy for confirmed HIT patients. Eleven studies reported the occurrence of thrombotic events in HIT patients. Seven studies reported the incidence of bleeding events in HIT patients. Fourteen studies reported the mortality of HIT patients.

**Table 1:** Details of the primary and secondary outcomes

Study	HIT (HIT/population)	Suspected HIT (suspected HIT/population)	Diagnostic algorithms	Immunoassay (method and cutoff/outcomes)	Functional assay	Diagnostic mode
Glick,2015 [8]	1/119	23/119	4Ts of suspected HIT: 5 (IQR, 4–6)	ELISA OD ≥ 0.4/3(+)	SRA	SRA positive or ELISA OD ≥ 2.0
Kutleˇsa,2017 [9]	3/40	n.a.	n.a	ELISA	n.a.	ELISA
Kimmoun, 2018 [10]	21/5797	39/5797	4Ts: Excluded HIT 5.0 (5.0–6.0), Confirmed HIT 5.0 (5.0–6.0)	ELISA/OD: Excluded HIT 1.253 [1.007–1.770], Confirmed HIT 1.080 [0.695–1.684]	PAT or HIPA and/or SRA	HIT-positive anti-PF4/heparin antibodies and at least one positive functional assay
Pabst,2019 [11]	14/455	n.a.	n.a.	ELISA OD > 0.4	SRA	SRA
Vayne,2019 [12]	3/57	2/57	n.a.	ELISA OD ≥ 0.4 /PF4-specific IgG, A, and M Abs 29(+);IgG Abs 17(+) high titers of 3 HIT patients are 1.8, 2.4, 2.8.	SRA and PF4-SRA	SRA and PF4-SRA
Arachchillage,2020 [13]	19/298	63/298	4Ts of HIT patients 4 (range:3-7) 3 low probability, 12 intermediate probability, 4 high probability in HIT patients	1. LIA(Hemosil HIT-Ab) > 1.0 U/mL/21(+) 2. ELISA OD > 0.5/19(+) 3. Hemosil AcuStar HIT-IgG (PF4-H) > 1.0 U/mL	n.a.	1. All three tests(+) 2. LIA(+) and ELISA OD > 1.0
Kataria,2020 [14]	9/473	47/473	n.a.	ELISA OD ≥ 1.0/11(+) (7 SRA+,4 SRA-) SRA(-) 0.59 ± 0.75 SRA(+) 1.58 ± 0.65	SRA	SRA
Sullivan,2020 [15]	2/134	39/134	4Ts:15 patients ≥ 4 HEP:5 patients ≥ 2 LLL:15 patients ≥ 2	ELISA OD > = 0.4/6(+) OD, median [IQR] ELISA positive 1.41 [0.63–2.51] ELISA negative 0.11 [0.09–0.16]	SRA	SRA
Wood,2020 [16]	10/131	n.a.	n.a.	PF4/Of the 10 HIT patients 8 patients OD > 1.0, Median 1.51 [1.02–2.51]	n.a.	Positive platelet factor 4 (PF4)
Mazzeffi,2021 [17]	2/20	6/20	6 (30%) patients 4Ts > 4	ELISA OD > 0.5/4(+)	SRA	SRA
Giuliano,2021 [18]	4/144	12/144	n.a	PF4	SRA	PF4 + SRA
Zaaqoq,2022 [19]	15/417	162/417	n.a.	ELISA OD ≥ 0.4/ 42(+), OD 0.63 ± 0.82; SRA Negative 28 (28.3%) +, OD 0.41 ± 0.52; SRA positive 93.3% positive, OD 2.10 ± 0.90	SRA	SRA
Arachchillage,2022 [20]	16/152	n.a.	n.a.	ELISA or Hemosil AcuStar HIT-IgG	platelet aggregation assay	ELISA or Hemosil AcuStar HIT-IgG or platelet aggregation assay
Hanna, 2022 [21]	n.a.	n.a.	4Ts/2 (20%) low probability, 4 (40%) intermediate probability, 4 (40%) high probability in HIT patients	ELISA OD > 0.4/HIT patients median 1.37 (0.83–2.0)	SRA	Positive SRA; If an SRA was unavailable, a positive anti-PF4 ELISA result was used for diagnosis of HIT
Kram, 2022 [22]	3/105	8/105	4Ts/All ECMO patients: median (IQR) 2 (2,4); high 3 (2.9%), Intermediate 26 (24.8%) HEP/All ECMO patients: median (IQR) -1 (-4, 2); high: 8 (7.6%) Intermediate 13 (12.4%)	ELISA OD > 0.4/12 (+)	SRA	SRA
Lubnow, 2022 [23]	16/507	81/507	4Ts	ELISA (PF4-IgG, Immucor GTI) and CLIA (HemosIL AcuStar HITIgG, IL) was used	HIPA	4T-Score ≥ 4, positive ELISA and positive HIPA test
Mang,2022 [24]	1/41	n.a.	n.a.	ELISA/7(+)	HIPA	HIPA
Kutleša, 2023 [25]	44/112	n.a.	n.a.	ID-PaGIA Heparin/PF4 Antibody Test and ELISA	n.a.	ELISA after the ID-PaGIA Heparin/PF4 Antibody Test was positive
Lüsebrink,2023 [26]	13/373	53/373	4Ts/suspicion HIT 4 (4,5) excluded HIT 4 (3,5) confirm HIT 5 (4,6)	Anti-PF4/heparin antibody	HIPA/SRA/PAT	The combination of positive anti-PF4/heparin antibodies with at least one positive functional assay, i.e., SRA, HIPA, and/or PAT

Study	HIT patients platelet count (mean ± SD/median (IQR)) × 109/L	Alternative anticoagulation	Monitoring targets	Thrombosis event	Bleeding event (bleeding event/HIT	Mortality (death/HIT)
Glick,2015 [8]	Suspected HIT, Baseline 215 (98–277) nadir within first 3 d, 69 (22–126) the time of H-PF4 testing 43 (23–73)	Argatroban	n.a.	n.a.	n.a.	n.a.
Kutleˇsa,2017 [9]	n.a.	Fondaparinux 2.5 mg daily	n.a.	n.a.	n.a.	3/3
Kimmoun, 2018 [10]	Baseline 173.0 (129.0–204.0) Platelets at HIT suspicion, 41.0 (23.0–61.0)	Argatroban, Bivalirudin, Danaparoid sodium, Fondaparinux, Replacement with a heparin-free circuit	n.a.	7/21	12/21	7/21
Pabst,2019 [11]	at days of heparin discontinuation 59.79 ± 32.238 at 14 days after discontinuation of heparin 280.2 ± 178.29	Argatroban 2mcg/kg/min	APTT 50–60 s	n.a.	n.a.	3/14
Vayne,2019 [12]	2 HIT suspected patients PLT declined 49 and 32%, respectively.	n.a.	n.a.	1/3	n.a.	3/3
Arachchillage,2020 [13]	Baseline 153.11 ± 49.47, nadir44.37 ± 14.28, % decrease in platelet count at the diagnosis of HIT 68.89 ± 7.06 platelet recovery of greater than 50% above the nadir, 96 h (48–120 h)	Argatroban 0.2 µg/kg/min	APTT 48–78 s	17/19	1/19	6/19
Kataria,2020 [14]	Baseline, 169.9 ± 79.5 nadir, 27.1 ± 14.1 PLT percentage fall, 79.8 ± 14.2	Fondaparinux, argatroban, or bivalirudin	n.a.	2/9	5/9	4/9
Sullivan,2020 [15]	Baseline, ELISA positive 177.5 (152.0-250.5) Percent platelet decline, ELISA positive 74.0 (56-84.3)	Argatroban Bivalirudin	n.a.	n.a.	n.a.	n.a.
Wood,2020 [16]	n.a.	Bivalirudin	n.a.	n.a.	n.a.	n.a.
Mazzeffi,2021 [17]	n.a.	DTIs	n.a.	1/2 (cannula)	n.a.	0/2
Giuliano,2021 [18]	ECMO DAY1:146 ± 98	Bivalirudin	APTT 50–65 s	1/4	4/4	3/4
Zaaqoq,2022 [19]	Baseline,223.5 ± 69.1; Day of testing, 50.4 ± 31.3 % ≥50% platelet drop by testing, 86.7%	n.a.	n.a.	3/15	n.a.	8/15
Arachchillage,2022 [20]	n.a.	n.a.	n.a.	10/16	n.a.	3/16
Hanna, 2022 [21]	Minimum value 47 (36.8–65.3)×10^3^/µL prior to PF4 order 47 (37.8–65.3)×10^3^/µL All HIT patients increased at least 25% after bivalirudin initiation	Bivalirudin	APTT 46–65 s	6/12	10/12	6/12
Kram, 2022 [22]	n.a.	n.a.	n.a.	n.a.	n.a.	n.a.
Lubnow, 2022 [23]	Start ECMO,/nL, 241 (135–334) nadir,/nL 46 (32–81)	Argatroban	HIT-suspicion APTT 50 s Confirm HIT APTT 60 s	10/16	6/16	5/16
Mang,2022 [24]	n.a.	Argatroban	n.a.	n.a.	n.a.	n.a.
Kutleša, 2023 [25]	n.a.	Fondaparinux	n.a.	n.a.	n.a.	36/44
Lüsebrink,2023 [26]	Admission 176 (112, 250); at the beginning of VA-ECMO 137 (117, 188); day 3 of VA-ECMO 82 (43, 99); day 7 of VA-ECMO 82 (65, 156); day 14 of VA-ECMO 200 (142, 260); Minimum during heparin therapy 50 (26, 65) Maximum during heparin therapy 176 (124, 206).	Argatroban	n.a.	4/13	4/13	5/13

### Risk of bias and study quality

Because all studies eligible for inclusion were retrospective studies, we evaluated study quality using the Newcastle‒Ottawa Quality Assessment Scale adapted for cross-sectional studies, which showed a high level of quality in 10 studies with a score greater than 7/10. The other 9 studies achieved a score of 5/10. The summary of the risk of bias is reported in Supplementary Table 2. Funnel plots for all the included outcomes are shown in Supplementary Figs. 3–6.

### Primary outcomes

#### HIT

**Fig. 2:**
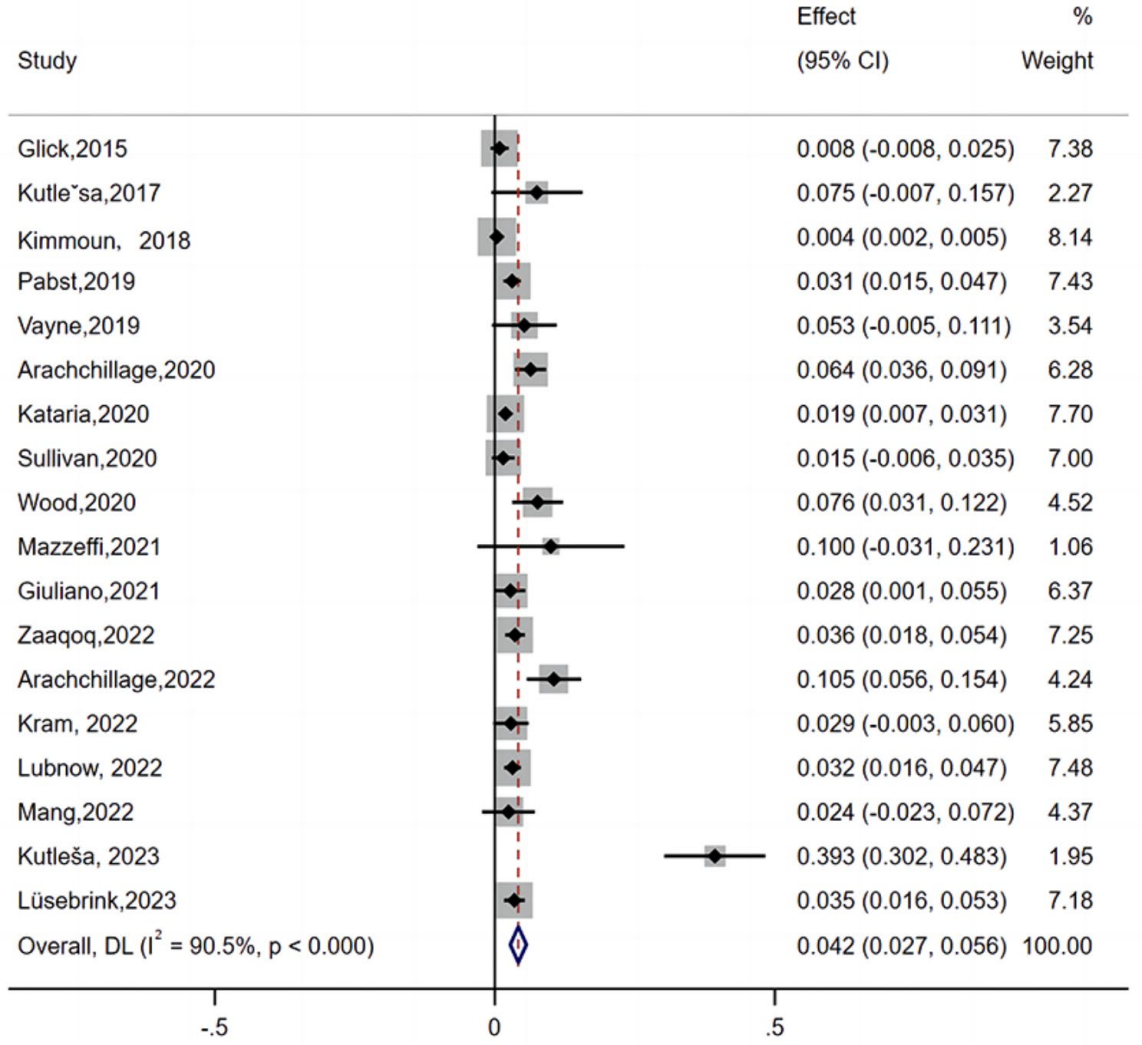
Forest plot showing meta-analysis of the incidence of HIT in ECMO-supported patients

A total of 18 studies reported the number of patients with HIT during ECMO support [8–20, 22–26]. The lowest incidence of HIT was 0.4%, while the highest was 39.3% [10, 25]. According to the random-effects analysis, the pooled incidence of HIT on ECMO was 4.2% (95% CI: 2.7–5.6, I2 = 90.5%) (Fig. 2).

The pooled incidence of HIT on ECMO was 4.2% (95% CI: 2.7–5.6, I^2^ = 90.5%). The black diamonds indicate individual estimates, and the black lines indicate individual 95% CIs. The gray squares represent the individual study weights. The vertical red dashed line indicates the pooled estimate. The vertical axis of the white diamond indicates a pooled estimate, whereas the horizontal axis indicates a pooled 95% CI.

#### Suspected HIT

**Fig. 3:**
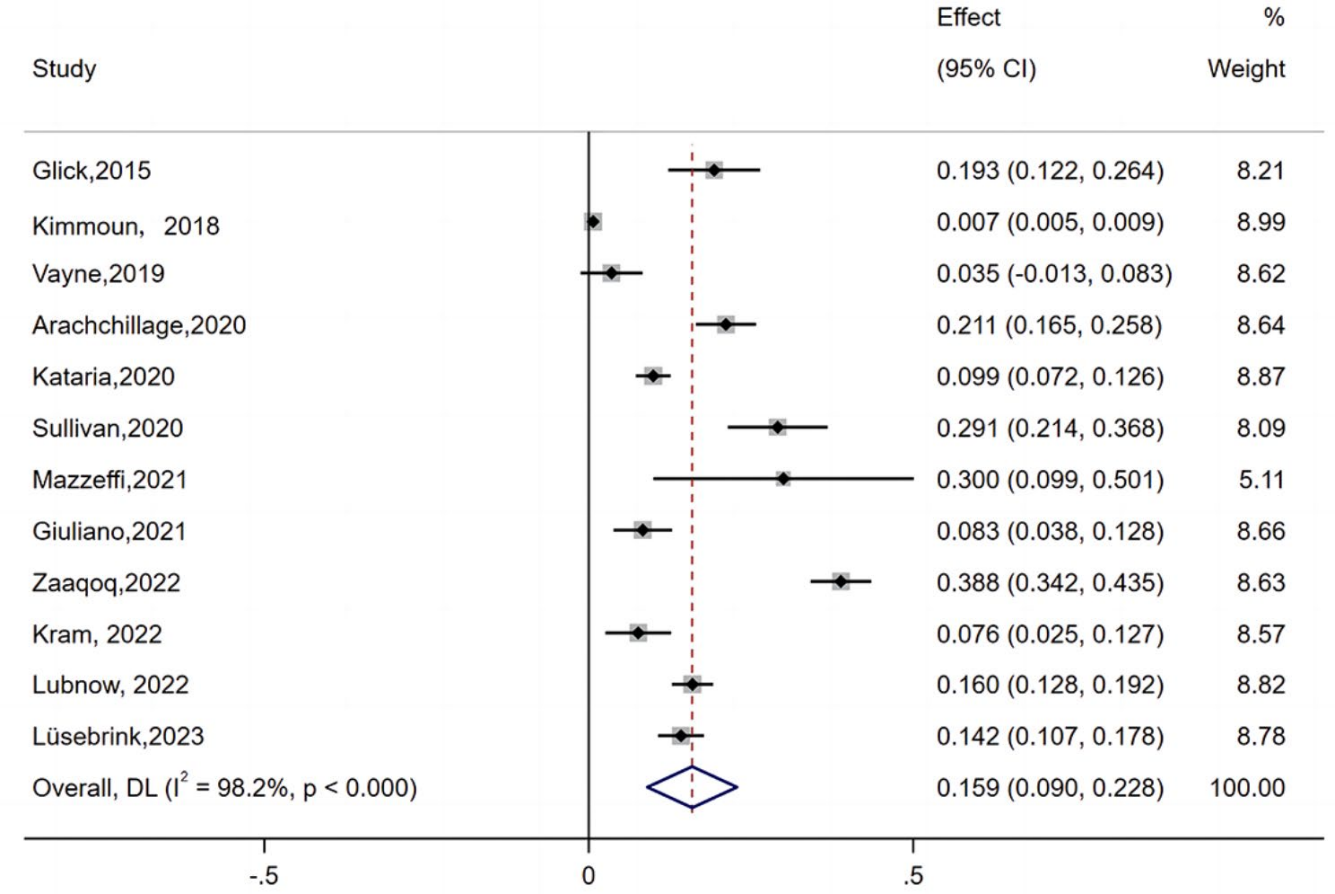
Forest plot showing the meta-analysis of the incidence of suspected HIT in ECMO-supported patients

In most institutions, HIT is suspected when the patient has a 4Ts score ≥ 4, a decrease in the PLT over 50%, or thrombosis after receiving heparin. Twelve of 19 studies presented the occurrence of suspected HIT [8, 10, 12–15, 17–19, 22, 23, 26]. The lowest incidence of suspected HIT is 0.7%, and the highest incidence of suspected HIT is 38.8% [10, 19]. There was severe heterogeneity (I^2^ = 98.2%). A random-effects model was used to analyze the data. As shown in Figs. 3 and 15.9% (95% CI: 9.0-22.8) of patients who were supported by ECMO were suspected of having HIT.

The pooled incidence of HIT on ECMO was 15.9% (95% CI: 9.0-22.8, I^2^ = 98.2%). The black diamonds indicate individual estimates, and the black lines indicate individual 95% CIs. The gray squares represent the individual study weights. The vertical red dashed line indicates the pooled estimate. The vertical axis of the white diamond indicates a pooled estimate, whereas the horizontal axis indicates a pooled 95% CI.

### Secondary outcomes

#### Diagnostic algorithms

Nine studies [8, 10, 13, 15, 17, 21–23, 26] described the diagnostic algorithms used to determine the likelihood of HIT in ECMO patients. The 4Ts score (score 0–3 = low HIT probability; score 4–5 = intermediate HIT probability; score 6–8 = high HIT probability) was used in all 9 studies. The 4T score of 11.2-30% of patients on ECMO was ≥ 4 [15, 17]. According to two studies [13, 21], 80-84.5% of confirmed HIT patients who received ECMO support had a 4Ts ≥ 4. The median 4Ts in HIT patients was 5 in 3 studies [8, 10, 26]. The HEP (HIT Expert Probability) score was used in two studies [15, 22], and 3.7% (5/134) and 20% (21/105) of ECMO patients were considered HEP positive. Only one study reported the outcome of LLL (Lilo-Le Louet score); 11.2% (15/134) of ECMO patients were LLL positive [15]. The details are shown in Table 1.

#### Immunoassay

Immunoassays detect anti-heparin/platelet Factor 4 (PF4) antibodies, which are ordered assist in the diagnosis of HIT. At least one kind of immunoassay was performed in all studies. As shown in Table 1, ELISA was used as the immunoassay in the majority of studies. The cutoff of the ELISA optical density (OD) value was 0.4 in 7 studies [8, 11, 12, 15, 19, 21, 22], 0.5 in 2 studies [13, 17], and 1.0 in 1 study [14]. The mean/median ELISA OD value of HIT patients ranges from 1.080 to 2.10 [10, 19]. Hemosil AcuStar HIT-IgG was used in 3 studies [13, 20, 23], and the cutoff was 1.0 in Arachchillage et al.’s study [13].

#### Functional assay

Fifteen studies performed functional assays to confirm HIT. Among them, 12 studies [8, 10–12, 14, 15, 17–19, 21, 22, 26] used the serotonin release assay (SRA) as the confirmatory test for HIT. A heparin-induced platelet activation assay (HIPA) was used in 4 studies [10, 23, 24, 26], and a platelet aggregation test (PAT) was performed in 3 studies [10, 20, 26].

#### Diagnostic mode

**Fig. 4:**
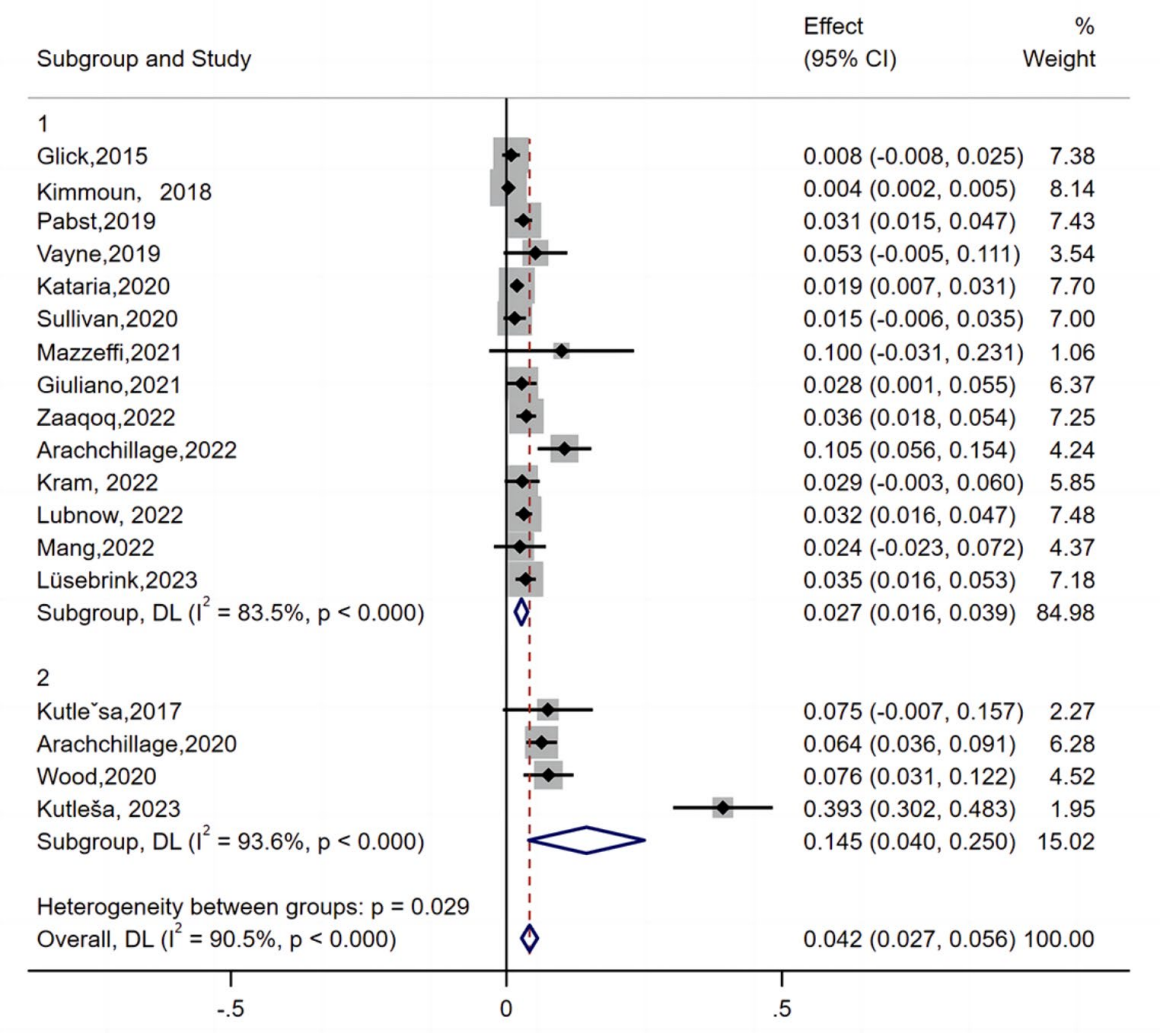
Subgroup analysis of different diagnostic methods

Functional assays were performed in 15 studies as the confirmatory test for HIT [8, 10–12, 14, 15, 17–24, 26]. Four studies confirmed HIT by immunoassay [9, 13, 16, 25]. Glick et al. reported that HIT can be confirmed by SRA positivity or an ELISA with an OD ≥ 2.0. Arachchillage et al. [13] performed a latex immunoturbidimetric assay (LIA, Hemosil HIT-Ab), ELISA, and Hemosil AcuStar HIT-IgG. HIT was confirmed when all three tests were positive or LIA positive and when the ELISA OD was > 1.0. To compare different diagnostic methods, we conducted subgroup analyses. As shown in Fig. 4, in the subgroup diagnosis by functional assay, the pooled incidence of HIT on ECMO was 2.7% (95% CI: 1.6–3.9). However, in the subgroup diagnosis by immunoassay, the pooled incidence was 14.5% (95% CI: 4–25).

Subgroup 1: Diagnosis by functional assay; subgroup 2: Diagnosis by immunoassay.

The pooled incidence of HIT on ECMO was 2.7% (95% CI: 1.6–3.9, I^2^ = 83.5%) in subgroup 1. The pooled incidence was 14.5% (95% CI: 4–25, I^2^ = 93.6%) in subgroup 2.

The black diamonds indicate individual estimates, and the black lines indicate individual 95% CIs. The gray squares represent the individual study weights. The vertical red dashed line indicates the pooled estimate. The vertical axis of the white diamond indicates a pooled estimate, whereas the horizontal axis indicates a pooled 95% CI.

#### Platelet count

Platelet counts of HIT patients were described in 11 studies [8, 10–15, 18, 19, 21, 23, 26]. The mean/median PLT at HIT suspicion ranged from 41.0 to 59.8 × 10^9^/L. The nadir PLT can reach 27.1–50 × 10^9^/L [14, 26]. The lowest decrease in PLT was 32% [12], while the greatest decrease was 79.8% [14]. Two studies [8, 10] showed that the PLT did not decrease further after heparin discontinuation. In Glick et al.’s study [8], the difference in the PLT nadir between patients suspected of having HIT and those not suspected of having HIT was statistically significant. However, in the group of patients suspected of having HIT, several studies confirmed that there was no significant difference in the PLT nadir or percentage of falls between patients who were confirmed to have HIT and patients who ultimately had HIT excluded by laboratory tests [14, 15, 19, 23, 26].

#### Alternative anticoagulation and monitoring target

Among a total of 19 studies, 15 studies [8–11, 13–18, 21, 23–26] reported alternative anticoagulation therapy for confirmed HIT patients. Argatroban was used in 9 studies [8, 10, 11, 13–15, 23, 24, 26]. Bivalirudin was used in 6 studies [10, 14–16, 18, 21]. Four studies used fondaparinux [9, 10, 14, 25]. Danaparoid was used in one study [10]. In addition, the replacement of a heparin-free circuit of ECMO was reported in one study [10]. The monitoring targets of alternative anticoagulation agents were identified in 5 studies. Three studies used argatroban with monitoring targets of activated partial thromboplastin time (APTT) of 48–78 s [13], 50–60 s [11] and [23]60 s. In 2 studies, heparin was transferred to bivalirudin, which was monitored by APTT 50–65 s [18] and 46–65 s [21]. (Table 1)

#### Thrombotic event

**Fig. 5:**
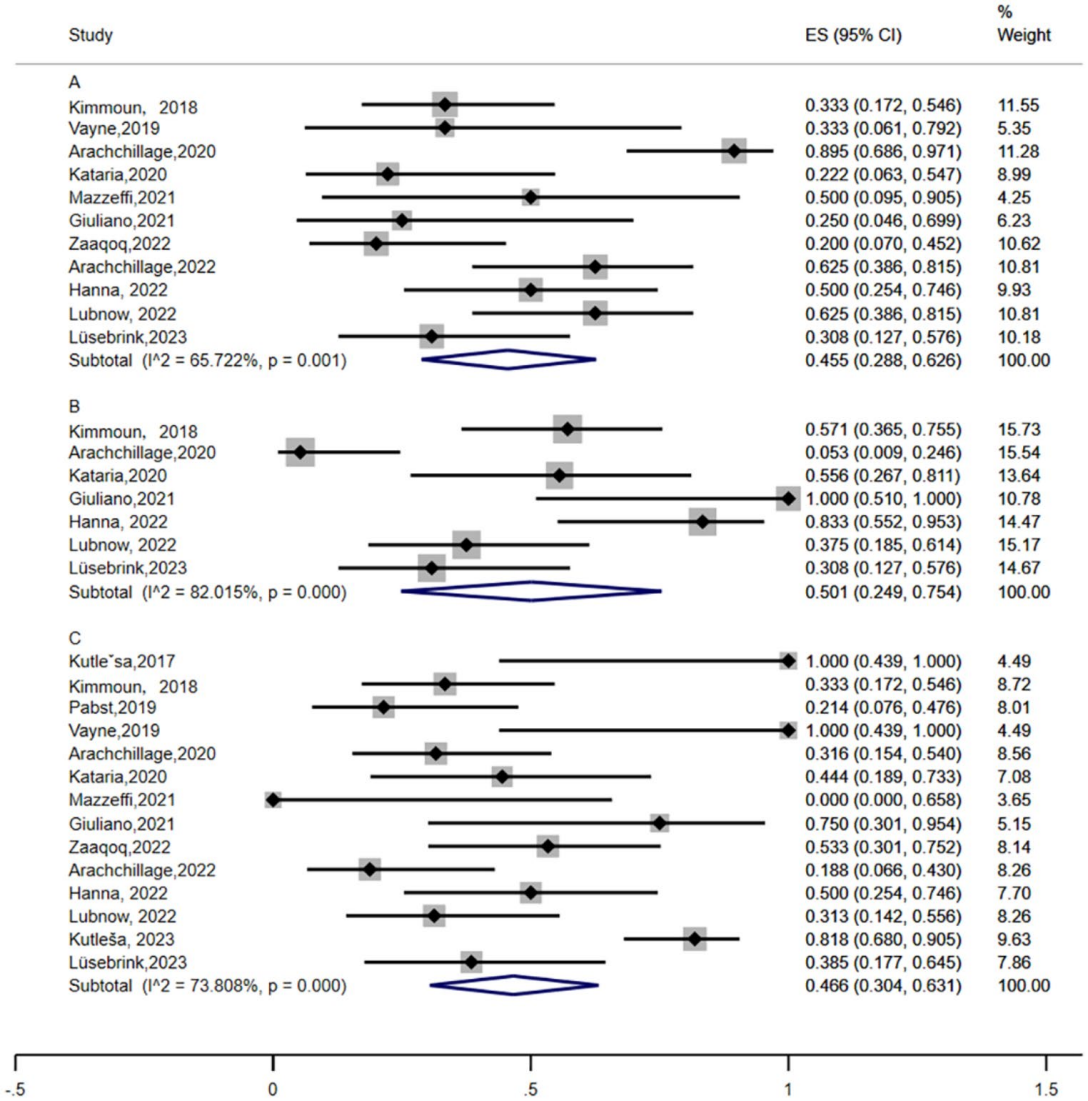
The incidence thrombotic events (A), bleeding events (B), and mortality (C) in confirmed HIT patients who received ECMO support

**Fig. 6:**
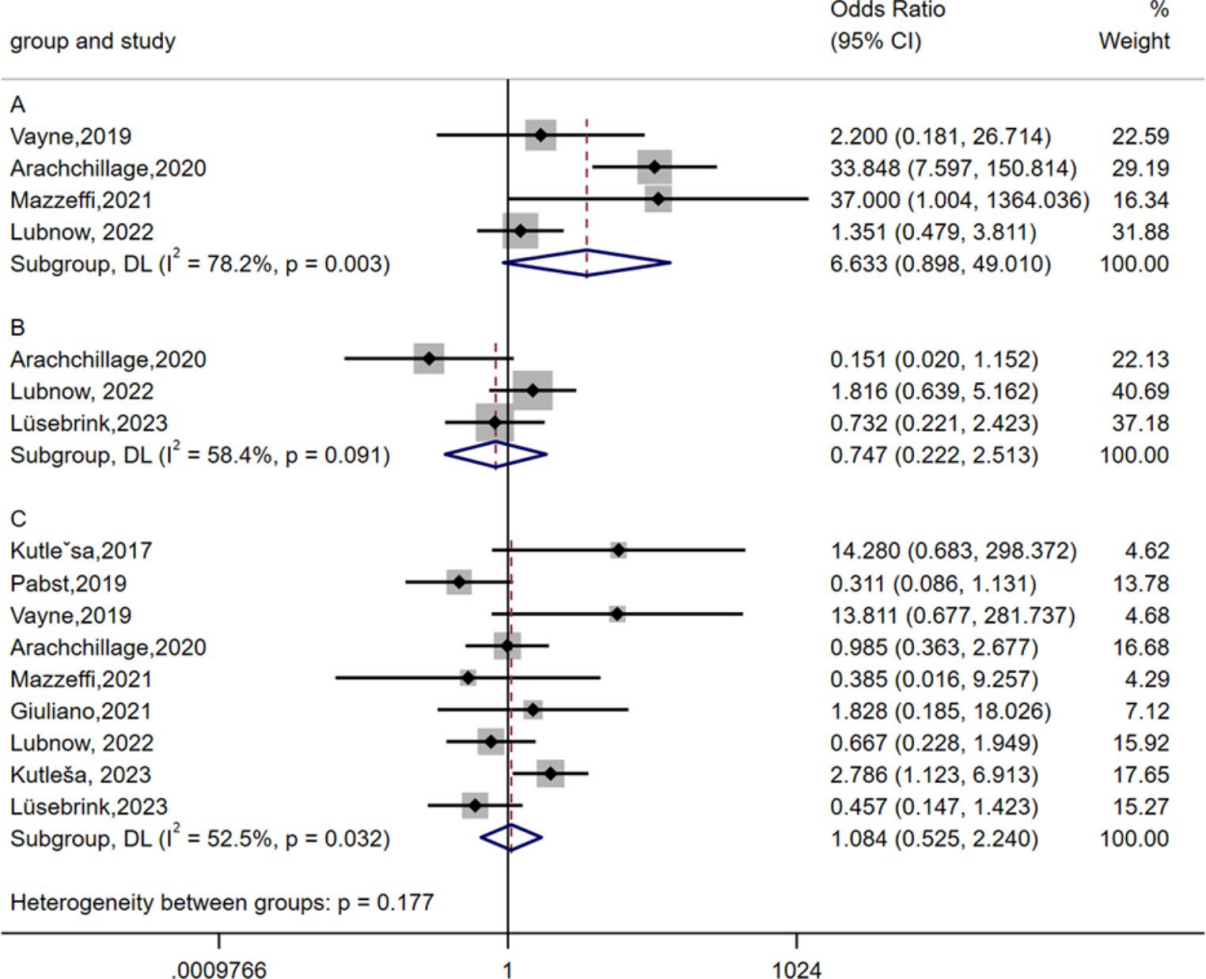
Forest plot showing the meta-analysis of the odds ratio of thrombosis formation **(A)**, bleeding **(B)**, and death **(C)** in confirmed HIT patients who received ECMO support

HIT can be associated with thrombosis (heparin-induced thrombocytopenia and thrombosis, HITT). Thrombotic events were reported in 11 studies [10, 12–14, 17–21, 23, 26]. The meta-analysis was performed using random effects analysis. As shown in Fig. 5 (A), the pooled incidence of thrombotic event in HIT patients was 45.5% (95% CI: 28.8–62.6, I^2^ = 65.722%). Thrombotic events on ECMO manifested as limb ischemia, ischemic stroke, intracardiac thrombus, deep vein thrombosis (DVT), and pulmonary embolus (PE). The odds ratio of thrombosis formation in HIT patients compared with non-HIT patients was 6.633 (95% CI: 0.898–49.010, I^2^ = 78.2%) (Fig. 6 (A)).

A. Forest plot showing the meta-analysis of the incidence of thrombotic events in confirmed HIT patients who received ECMO support. The pooled incidence of thrombotic events in HIT patients was 45.5% (95% CI: 28.8–62.6, I^2^ = 65.722%). B. Forest plot showing the meta-analysis of the incidence of bleeding events in confirmed HIT patients who received ECMO support. The pooled incidence of bleeding events in HIT patients was 50.1% (95% CI: 24.9–75.4 I^2 ^= 82.015%). C. Forest plot showing the meta-analysis of mortality in confirmed HIT patients who received ECMO support. The pooled mortality of HIT patients was 46.6% (95% CI: 30.4–63.1, I^2^ = 73.808%). The black diamonds indicate individual estimates, and the black lines indicate individual 95% CIs. The gray squares represent the individual study weights. The vertical red dashed line indicates the pooled estimate. The vertical axis of the white diamond indicates a pooled estimate, whereas the horizontal axis indicates a pooled 95% CI.

#### Bleeding event

Seven studies [10, 13, 14, 18, 21, 23, 26] reported the incidence of bleeding events in HIT patients. The incidence of this disease ranges from 5.3-100% [13, 18]. A random-effects model was used to analyze the data, and the pooled incidence of bleeding events in HIT patients was 50.1% (95% CI 24.9–75.4 I^2^ = 82.015%) (Fig. 5(B)). The location of bleeding included the surgical site, central nervous system, and gastrointestinal tract. Severe bleeding can lead to patient death. The odds ratio of bleeding in HIT patients compared with non-HIT patients was 0.747 (95% CI: 0.222–2.513, I^2^ = 58.4%) (Fig. 6 (B)).

A. The odds ratio of thrombosis formation in HIT patients compared with non-HIT patients was 6.633 (95% CI: 0.898–49.010, I^2^ = 78.2%).

B. The odds ratio of bleeding in HIT patients compared with non-HIT patients was 0.747 (95% CI 0.222–2.513, I^2^ = 58.4%).

C. The odds ratio of death in HIT patients compared with non-HIT patients was 1.084 (95% CI 0.525–2.240, I^2^ = 52.5%).

The black diamonds indicate individual estimates, and the black lines indicate individual 95% CIs. The gray squares represent the individual study weights. The vertical red dashed line indicates the pooled estimate. The vertical axis of the white diamond indicates a pooled estimate, whereas the horizontal axis indicates a pooled 95% CI.

#### Mortality

Thirteen of these studies reported the mortality of HIT patients [9–14, 17–19, 21, 23, 25, 26]. A random-effects model was used for the meta-analysis, and the pooled mortality of HIT patients was 46.6% (95% CI 30.4–63.1, I^2^ = 73.808%) (Fig. 5(C)). The odds ratio of death in HIT patients compared with non-HIT patients was 1.084 (95% CI 0.525–2.240, I^2^ = 52.5%) (Fig 6 (C)).

### Sensitivity analysis

The results of the sensitivity analysis in which one study was omitted at a time to identify influential studies are shown in Supplementary Figs. 1–2.

## Discussion

In previous studies, the incidence of HIT ranged from 0.1 to 5.0% in patients who received heparin for various indications [27–30]. In particular, mechanical circulatory support systems (such as ECMO) carry the potential for HIT due to the need for systemic anticoagulation [31]. Arachchillage et al. [13] reported that the frequencies of HIT in patients receiving ECMO and CPB were 6.4% and 0.6%, respectively. In this systematic review and meta-analysis, the incidence of HIT in ECMO-supported patients was 4.2% (95% CI: 2.7–5.6, I^2^ = 90.5%). The higher incidence of HIT on ECMO may be due to the following reasons: (1) Heparin/platelet Factor 4 (PF4) complex formation requires appropriate PF4-to-heparin ratios. Patients on ECMO could achieve stoichiometrically optimal PF4-to-heparin ratios due to a bolus of heparin at initiation and following systematic UFH infusion [13, 17]. (2) Persistent inflammation/infection in ECMO patients also enhances the immune response, leading to PF4-heparin complex formation [13, 17].

The incidence of suspected HIT was 15.9% (95% CI: 9.0-22.8) in our study. The most commonly used tool to evaluate the probability of HIT is the 4Ts score [32]. The score considers (1) PLT count or reduction in PLT count, (2) the time after heparin initiation that thrombocytopenia occurs, (3) thrombosis or other complications, and (4) a possible reason for the loss of platelets except for HIT. However, the ECMO system can also lead to thrombocytopenia and thrombosis formation. This makes it difficult for clinicians to use the 4Ts score to predict HIT on ECMO. There are also less commonly used tools, such as the LLL score and HEP score. The HEP score reflects the PLT count and clinical manifestations using an integer scale ranging from − 3 to + 3 to improve the specificity and sensitivity of the identification of HIT [33]. The LLL score focuses on patients who underwent CPB [34]. In a study conducted by Sullivan and colleagues [15], the 4T score showed limited effectiveness, as evidenced by a low positive predictive value (PPV) of 21.4%, along with 50% sensitivity and 66.7% specificity. Although the HEP test increased the specificity to 84.8%, it resulted in decreased PPV and sensitivity, both at 16.7%. Similarly, LLL scores were also found to be of limited use in this particular study by Sullivan et al. In a recent study [22] conducted on 299 patients on mechanical circulatory support devices (MCSs), the area under the curve (AUC) of the 4T score and the HEP score were 0.82 and 0.78, respectively, in ECMO or ventricular assist device (VAD)-supported patients. Both the 4Ts score and the HEP score demonstrated a low PPV of 23% [22]. In summary, all three tools had limited utility in predicting the probability of HIT in patients receiving ECMO support. The most suitable tool for clinicians to judge the probability of HIT in patients supported by ECMO remains to be studied.

After suspicion of HIT, a laboratory test will be performed to diagnose HIT. There are 2 classes of laboratory tests: immunoassays and functional assays. ELISA is the most commonly used immunological assay to rule out HIT after suspicion. However, the appropriate cutoff value is still controversial, and an improper threshold may lead to overdiagnosis. Most commercial kits recommend 0.4 as the cutoff value. According to the report by Kataria et al. [14] focused on ECMO patients, a specificity of 89% and a negative predictive value (NPV) of 95% were achieved with ELISA using OD ≥ 1.0 as the cutoff. There was high discrimination, with an AUROC (95% CI) of 0.92 (0.85-1.00). A study by Zaaqoq et al. [19] reported that after changing the cutoff value of the ELISA from OD ≥ 0.4 to 1.2, both specificity (from 71.7 to 90.9%) and accuracy (from 76.4 to 89.5%) improved, with only a limited impact on sensitivity (80%). To rapidly and automatically detect HIT antibodies, several other immunoassays have been developed, such as chemiluminescent immunoassays (e.g., Hemosil AcuStar HIT-IgG) [35].

Most of the institutions in our study performed the SRA as the confirming test for HIT. SRA has a high sensitivity and specificity for HIT diagnosis [36] and is regarded as the gold standard. However, due to its complexity and radioactivity, this test is available only in selected laboratories, which may lead to a prolonged turnaround time. Therefore, the reliance on SRA results may delay subsequent treatment. According to our subgroup analysis, the pooled incidence of HIT in ECMO patients diagnosed by immunoassays (14.5%, 4 studies) was significantly greater than that in patients diagnosed by functional assays (2.7%, 14 studies). We suggest that relying on immunoassay results for the diagnosis of HIT may lead to overdiagnosis. Therefore, it is crucial to develop functional tests that are fast, convenient, and accurate.

Thrombocytopenia is the most significant manifestation in HIT patients. Patients develop mild to moderate absolute thrombocytopenia (PLT between 50 and 70 × 10^9^/L) or relative thrombocytopenia (a decrease in the PLT of 30–50%) [37]. However, thrombocytopenia is also common in ECMO-supported patients. According to a systematic review and meta-analysis by Jiritano et al. [4], the pooled prevalence of thrombocytopenia in ECMO-supported patients was 21% (95% CI 12.9–29.0; 14 studies). The most important reason for the decrease in the PLT is consumption in the ECMO system. It has been demonstrated that platelet activation is enhanced by contact with artificial surfaces and high shear stress during ECMO treatment [4]. Other factors contributing to the decrease in PLT include blood dilution, sepsis, disseminated intravascular coagulation (DIC), and drug-induced thrombocytopenia in critically ill patients. Our research revealed that ECMO-supported patients with HIT can develop significant thrombocytopenia (PLT nadir 27.1–50 × 10^9^/L, percentage decrease of 32-79.8%). However, it is difficult to distinguish HIT-positive patients by PLT changes among suspected HIT patients. A former study [5] showed that the PLT continuously decreased over the first 2–7 days after ECMO initiation. However, the risk of rapid-onset HIT after ECMO initiation is low since antibodies to heparin-PF4 complexes take time. A decrease in the PLT usually occurs 5–14 days after heparin exposure [5, 37, 38]. Sokolovic et al. [39] showed that the PLT of excluded HIT patients steadily recovered after Day 5 of ECMO initiation, but the PLT of HIT-positive patients persistently decreased until Day 7. In a systematic review of 28 patients [40], the PLT nadir was achieved on Day 6 after the initiation of ECMO in HIT patients. According to previous studies [41, 42] focused on patients who underwent CPB, a biphasic PLT pattern may predict HIT in patients who underwent CPB. The PLT pattern of HIT patients on ECMO still needs to be studied.

During ECMO support, once patients have HIT, heparin exposure must be stopped, and alternative anticoagulation therapy should be started [5]. The choice of agent is determined according to the patient’s renal/liver function, ability to monitor anticoagulant effects, cost, bleeding risk, and clinicians’ experience. Argatroban, lepirudin, or danaparoid was recommended for patients with HIT or HITT according to the guidelines of the American College of Chest Physicians (ACCP) [43]. In our study, argatroban was the most common alternative for anticoagulation. Although bivalirudin is only recommended for patients who require urgent cardiac surgery in the ACCP guidelines, it was also widely used in ECMO-supported patients with HIT in our study. It is an attractive alternative for HIT patients because of its shorter half-life and lower effect on the international normalized ratio (INR) and nonhepatic metabolism [44]. According to a previous study [45], compared with argatroban, bivalirudin can reach the therapeutic APTT goal faster, with more APTT values within the therapeutic APTT goal, while the clinical outcomes are similar. Moreover, according to the American Society of Hematology (ASH) guidelines, argatroban or bivalirudin was recommended for patients with critical illness [46]. Furthermore, our study showed 50.1% (95% CI: 24.9–75.4 I^2^ = 82.015%) of HIT patients developed bleeding events. HIT itself does not cause bleeding [7]. It was supposed to be caused by overanticoagulation treatment to prevent thrombosis. In addition, inappropriate transitions to alternative anticoagulation agents may also lead to bleeding events. Therefore, clinicians need to monitor these patients carefully during alternative anticoagulant therapy. It is important to define monitoring targets, which still need further study. The influence of the heparin coating circuit in HIT patients is unknown, but it was supposed that the agent is unlikely to spread to the bloodstream. The study of Pabst et al. [11] reported that although HIT patients did not experience a change in the non-heparin-coated circuit, the PLT recovered well.

HIT can paradoxically lead to a prothrombotic disorder. Eventually, serious adverse outcomes, including ischemic limb necrosis, pulmonary embolism, and acute myocardial infarction, may develop [27]. HIT patients who have thrombosis formation can be diagnosed with HITT [47]. Approximately 30% of HIT patients experience thrombosis formation [7]. A total of 25.3% of VV-ECMO-supported patients and 37.9% of VA-ECMO-supported patients experienced thrombosis, which was reported in the registry analysis of the ELSO [48, 49]. According to our meta-analysis, the incidence of thrombotic event in HIT-confirmed patients receiving ECMO support was 45.5% (95% CI: 28.8–62.6, I^2^ = 65.722%). The odds ratio of thrombosis formation in HIT patients compared with non-HIT patients was 6.633 (95% CI: 0.898–49.010). Although it is not statistically significant, it revealed greater incidence of thrombosis formation in HIT patients.

Our data revealed that 46.6% (95% CI: 30.4–63.1, I^2^ = 73.808%) of HIT patients who received ECMO support died. Our results revealed that mortality was similar between patients with confirmed HIT and HIT-negative patients(OR:1.084 (95% CI: 0.525–2.240)). The reasons for HIT patient death in our included studies were postoperative multiorgan failure, brain bleeding, and sepsis [11, 12].

### Limitations

The findings and interpretations of this meta-analysis and systematic review are limited by the quality of included studies and high heterogeneity. Our study was designed to analyse the incidence of HIT and adverse events by meta-analysis, which is vulnerable to publication bias leading to overestimation of incidence rates. The sample size of confirmed HIT patients was too small for further analysis of the incidence of thrombotic /bleeding events and mortality. Thus the results and interpretation must be interpreted with care.

## Conclusion

According to our study, 4.2% of patients under ECMO support develop HIT. This leads to an increased risk of thrombosis in critically ill patients. Current clinical and laboratory diagnostic methods exhibit many shortcomings. Inappropriate diagnostic methods can easily lead to misdiagnosis of HIT. Further research and development of diagnostic algorithms and laboratory assays are warranted.

### Electronic supplementary material

Below is the link to the electronic supplementary material.

Supplementary Material 1

## Data Availability

No datasets were generated or analysed during the current study.

## Abbreviations

ECMO: Extracorporeal membrane oxygenation
HIT: Heparin-induced thrombocytopenia
OD: Optical density
ELISA: Enzyme-linked immunosorbent assay
SRA: Serotonin release assays
ELSO: Extracorporeal Life Support Organization
UFH: Unfractionated heparin
PLT: Platelet
CPB: Cardiopulmonary bypass
PRISMA: Preferred Reporting Items for Systematic Reviews and Meta-Analysis
ECLS: Extracorporeal life support
SD: Standard deviation
CI: Confidence interval
VV-ECMO: Venovenous extracorporeal membrane oxygenation
VA-ECMO: Venoarterial extracorporeal membrane oxygenation
HEP: HIT Expert Probability
LLL: Lilo-Le Louet score
HIPA: Heparin-induced platelet activation assay
PAT: Platelet aggregation test
LIA: Latex immunoturbidimetric assay
APTT: Activated partial thromboplastin time
HITT: Heparin-induced thrombocytopenia with thrombosis syndrome
DVT: Deep vein thrombosis
PE: Pulmonary embolus
PF4: Platelet Factor 4
PPV: Positive predictive value
MCS: Mechanical Circulatory Support Device
AUC: Area under curve
VAD: Ventricular assist device
DIC: Disseminated intravascular coagulation
ACCP: American College of Chest Physicians
INR: International normalized ratio
ASH: American Society of Hematology
