# Supplementary material for: Heparin-induced thrombocytopenia in extracorporeal membrane oxygenation-supported patients: a systematic review and meta-analysis
Source: Thromb J. 2024 Jun 28;22:55. doi: 10.1186/s12959-024-00624-5 (PMC11212165; doi:10.1186/s12959-024-00624-5)

Supplementary Table 1 Characteristics of studies

Study (author,year)	Study period	Type	Institution	Number of patients	age	VV-ECMO	VA-ECMO
Glick,2015[8]	2011.1-2013.6	Retrospective	Columbia University Medical Center,USA	119	N.a	65	54
Kutleˇsa,2017[9]	2009.10-2014.6	Retrospective,single-center study	Zagreb University Hospital for Infectious Diseases,Croatia	40	48.5 (33.3–61.5)	40	0
Kimmoun，2018[10]	2012-2016	Retrospective,multi-center	France	5797	N.a	0	5797
Pabst,2019[11]	2008.7-2017.7	Retrospective,single-center study	Heart and Vascular Institute, Penn State Health Milton S. Hershey Medical Center, Hershey, PA, USA	455	51.88 ±15.99	364	91
Vayne,2019[12]	2014.2-2018.1	Retrospective	the University Hospital in Tours,France	57	57range(24-76)	0	57
Arachchillage,2020[13]	2016.1-2018.4	Retrospective, single-center, observational cohort study	Royal Brompton and Harefield National Health Service ,UK	298	45.4± 15.6	156	142
Kataria,2020[14]	2012 - 2018	Retrospective,single-center study	Baylor University Medical Center, Dallas, TX,USA	473	N.a	N.a	N.a
Sullivan,2020[15]	2009.2.1-2018.2.28	Retrospective, single-center, observational study	Rush University Medical Center,USA	134	N.a	N.a	N.a
Wood,2020[16]	2011.5-2018.1	Retrospective	University of Rochester Medical Center,USA	131	56 ±14	0	131
Mazzeffi,2021[17]	N.a.	Retrospective	University of Maryland Medical Center, Baltimore, MD, USA	20	58±9	0	20
Giuliano,2021[18]	2016.1-2019.7	Retrospective cohort study	Johns Hopkins Hospital,USA	144	55.3± 15.8	28	116
Zaaqoq,2022[19]	2011.9-2020.9	Retrospective	Medstar Washington Hospital Center, Washington, DC,	417	N.a	N.a	N.a

			USA				
Arachchillage,2022[20]	2020.3.1-2020.5.31	Retrospective	UK	152	47 (range23–65)	152	0
Hanna, 2022[21]	2015.1-2020.2	Single-center retrospective, observational study	Cleveland Clinic	12	58 (54.5–63.3)	4	6(others 2)
Kram, 2022[22]	2013.7.1-2018.5.1	Retrospective	Duke University Medical Center	105	N.A.	N.A.	N.A.
Lubnow, 2022[23]	2006.1- 2016.12	Retrospective observational single centre study	University Hospital of Regensburg	507	N.A.	331	176
Mang,2022[24]	2020.3.1-2021.3.1	Retrospective	University Medical Centre, Saarland University	41	N.A.	41	0
Kutleša, 2023[25]	2020.2-2022.4	Retrospective cohort study	Zagreb University Hospital for Infectious Diseases,Croatia	112	N.A.	112	0
Lüsebrink,2023[26]	2013.1-2022.5	Retrospective single-center study	University Hospital of Munich	373	59(51,67)	0	373

Supplementary Table1 (continue)

Study(author, year)	ECMO duration	ECMO circuit	Inclusion criteria	Exclusion criteria	Monitoring Targets of Heparin
Glick,2015[8]	N.a	N.a	Age>18 years, no prior history of HIT, anticoagulated with UFH,ECMO duration>5 days	N.a	APTT 40-60s
Kutleˇsa,2017 [9]	192.0 (130.0-330.0)hours	Biomedicus cannulas,Medtronic I-4500 and Eurosets (Medolla, Italy)ADULT ECMO oxygenators and Medtronic (Dublin,Ireland) BIOtherm heat exchanger Biomedicus BP-80 pump	adult patients with ARDS treated with ECMO	N.A	ACT 170–180 s
Kimmoun , 2018[10]	N.a	N.a	Admitted to the ICU for the management of refractory cardiogenic shock under VA-ECMO for at least	Medical history of HIT, ongoing pregnancy, age < 18 years old,	APTT goal: excluded HIT 67.2 (66.9–70.0)s,confirmed HIT 65.25 (64.7–69.5)s Anti-Xa goal:excluded HIT 0.35

			3 days, be treated with UFH	admission for septic shock.	(0.2–0.45)UI/ml,confirmed HIT 0.35 (0.3–0.45)UI/ml
Pabst,2019[11]	9.46 ±10.87	Quadrox oxygenator (MAQUET Cardiovascular, Wayne, NJ, USA) Rotaflow® centrifugal pump (MAQUET Cardiovascular) or Centrimag® centrifugal pump (Thoratec Corporation, Pleasanton, CA, USA)	patients with ECMO	N.a	APTT of 50-60 s
Vayne,2019[12]	12[5-38]days	The Cardiohelp system was used in combination withthe HLS Set Advanced 7.0 oxygenator, both coated with Bioline (recombinant human albumin and heparin coating,Maquet, Getinge Group, Rastatt, Germany) and Medos Deltrastream (Rheoparin heating and cooling,heparinbased treatment, Medos Deltastream, Xenios, Germany)	treated with ECMO and heparin for at least five consecutive days	patients who did not have a sample available	APTT ratio 1.8-2.2
Arachchillage,2020[13]	170.4 hours (70–1,008 hr)	N.a	Received ECMO for more than 48 hours	1. Received ECMO for less than or equal to 48 hours; 2. Did not receive heparin at the initiation of ECMO; 3. Patients with a history of HIT .	Anti-Xa concentration was 0.2–0.3 U/mL for VV-ECMO and 0.3-0.5 U/mL for VA-ECMO
Kataria,2020[14]	N.a	N.a	age greater than or equal to 18 years, cannulation for ECMO support,initial anticoagulation with UFH.	N.a	APTT 40–60s for VV and 50–75 s for VA ECMO
Sullivan,2020[15]	N.a	N.a	Patients included were at least 18 years of age, on ECMO for any	preexisting heparin allergy or a equivocal anti-PF4 antibody	N.A.

			indication, and had an anti-PF4 antibody ordered while on ECMO.	result	
Wood,2020[16]	201.4 ±165.2hr	Cortiva BioActive Surface tubing (Medtronic, Minneapolis, MN). Uncoated circuits were used for patients who developed HIT.<br>Quadrox-i oxygenator (Maquet, Wayne, NJ) or EOS ECMO oxygenator (LivaNova, London, United Kingdom)<br>ROTAFLOW (Maquet, Wayne, NJ), CentriMagTM (Abbott, Chicago, IL) or TandemHeart (LivaNova, London, United Kingdom) pumps	adult VA-ECMO patients	VV ECMO and patients under 18 years of age,Adult patients who had active bleeding during the first 24 hours of VA-ECMO initiation	ACT 180-220s or APTT 54-71s
Mazzeffi,2021[17]	9 [7, 11]days	Rotaflow pump (Getinge Group, Wayne, NJ, USA) and Quadrox oxygenator (Getinge Group, Wayne, NJ, USA)	Adult patients on VA ECMO<br>for management of cardiogenic shock or decompensated congestive heart failure	N.a	APTT 60-80s
Giuliano,2021[18]	4.8 (2.6–10.0)days	N.a	all adult (> 18 yr) patients placed on ECMO	N.a	APTT 50-65s
Zaaqoq,2022[19]	N.a	N.a	subjects both on VV and VA ECMO ,have a confrmatory serotonin release assay	a HIT ELISA was ordered but for which there was no subsequent SRA	N.A.
Arachchillage,2022[20]	17. 5 days [(IQR) 11–30 days]	N.a	All consecutive adult patients (≥18 years) supported with VV ECMO for at least 48 h during the first wave of the COVID-19 pandemic in the UK	N.a	anti-Xa 0.2–0.3 IU/ml or equivalent (local) APTT<br>For patients with thrombosis:anti-Xa 0.5–0.7 IU/ml or equivalent APTT
Hanna, 2022[21]	328.5 (218.8–	Rotaflow (Maquet) pumps with Quadrox (Maquet) oxygenators and HLS (Maquet) cannulae (19–29 French).	least 18 years of age, supported on either V-A or	N.A.	APTT 49–67 s<br>(correlating to an anti-Xa 0.2-0.5

	502.1) h	For single-site cannulation, Avalon (Maquet) or Crescent (Medtronic) cannulae are utilized.	V-V ECMO for at least 48 h, treated with bivalirudin for acute HIT while requiring ECMO support, and had a positive SRA result.		IU/mL)
Kram, 2022[22]	N.A.	N.A.	1.Patients ≥18 years of age<br>2.Admitted to ICU with an MCS device in place for at least 24 hours<br>3.Present at the time of PF4 assay were included<br>4.Received LMWH or UFH within the 10 days prior to MCS device placement	1.Patients with a history of HIT within the previous 90 days<br>2.Preexisting durable MCS device<br>3.Plasmapheresis performed after heparin exposure<br>4.hepar ininitiation >1 day after MCS device placement	APTT 40-60s
Lubnow, 2022[23]	N.A.	PLS-system and Cardiohelp-system with heparin based Bioline-coating Gettinge / Maquet, Rastatt, Germany.<br>Hilite7000LT oxygenator + DP3 pump with heparin based Rheoparin-coating, Medos Medizintechnik, Stolberg, Germany; iLA-activve-system with heparin based Rheoparin-coating, Fresenius Medical Care / Xenios / Novalung, Heilbronn, Germany.<br>ECC.05 system with Phosphorylcholin-coating, Livanova / Sorin Group, Modena, Italy	severe respiratory failure receiving VV ECMO therapy and patients receiving VA ECMO for circulatory failure including those undergoing ECPR	N.A.	APTT 50s in VV ECMO and 60s in VA ECMO
Mang,2022[24]	11 ± 7 d	N.A.	patients with V-V ECMO for COVID-19 related respiratory failure	N.A.	APTT 45–55s
Kutleša, 2023[25]	N.A.	N.A.	all adult patients with confirmed COVID-19 ARDS that required ECMO treatment	N.A.	ACT 170-180

Lüsebrink,2023[26]	ALL 89 (39, 144)h HIT 176 (99, 215)h	N.A.		adult patients (≥18 years) with VA-ECMO support in the cardiac intensive care unit (ICU)	Patients <18 years, experiencing ongoing pregnancy, and who had a known history of HIT	APTT 60–80 s

Supplementary Table 2 Study quality

Study	Selection	Comparability	Outcome	Total
Glick,2015[8]	****	*	***	7
Kutleˇsa,2017[9]	***		***	6
Kimmoun，2018[10]	****	**	***	9
Pabst,2019[11]	***	*	***	7
Vayne,2019[12]	***	*	***	7
Arachchillage,2020[13]	***	*	***	7
Kataria,2020[14]	***	*	**	6
Sullivan,2020[15]	****	*	**	7
Wood,2020[16]	***		***	6
Mazzeffi,2021[17]	***		***	6
Giuliano,2021[18]	***		***	6
Zaaqoq,2022[19]	***	*	***	7
Arachchillage,2022[20]	**		***	5
Hanna, 2022[21]	**	*	***	6
Kram, 2022[22]	****	**	***	9
Lubnow, 2022[23]	***	**	***	8
Mang,2022[24]	**		***	5
Kutleša, 2023[25]	***		***	6
Lüsebrink,2023[26]	****	**	***	9

Supplementary figure 1 Sensitivity analysis of the incidence of HIT in ECMO patients

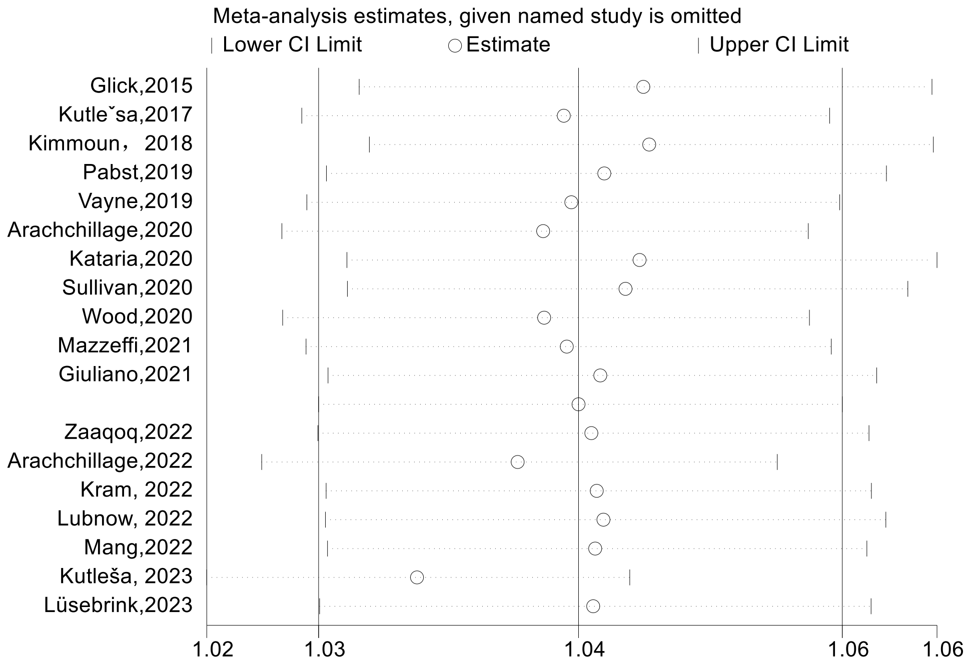

Supplementary figure 2: Sensitivity analysis of the incidence of suspected HIT in ECMO patients

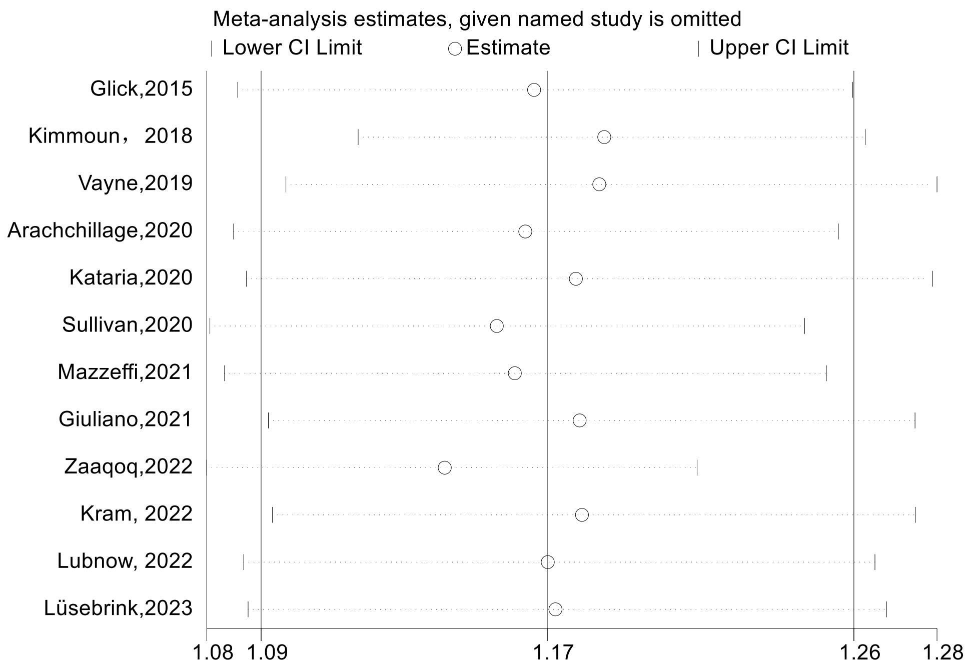

Supplementary figure 3: Funnel plot of the incidence of HIT in ECMO patients

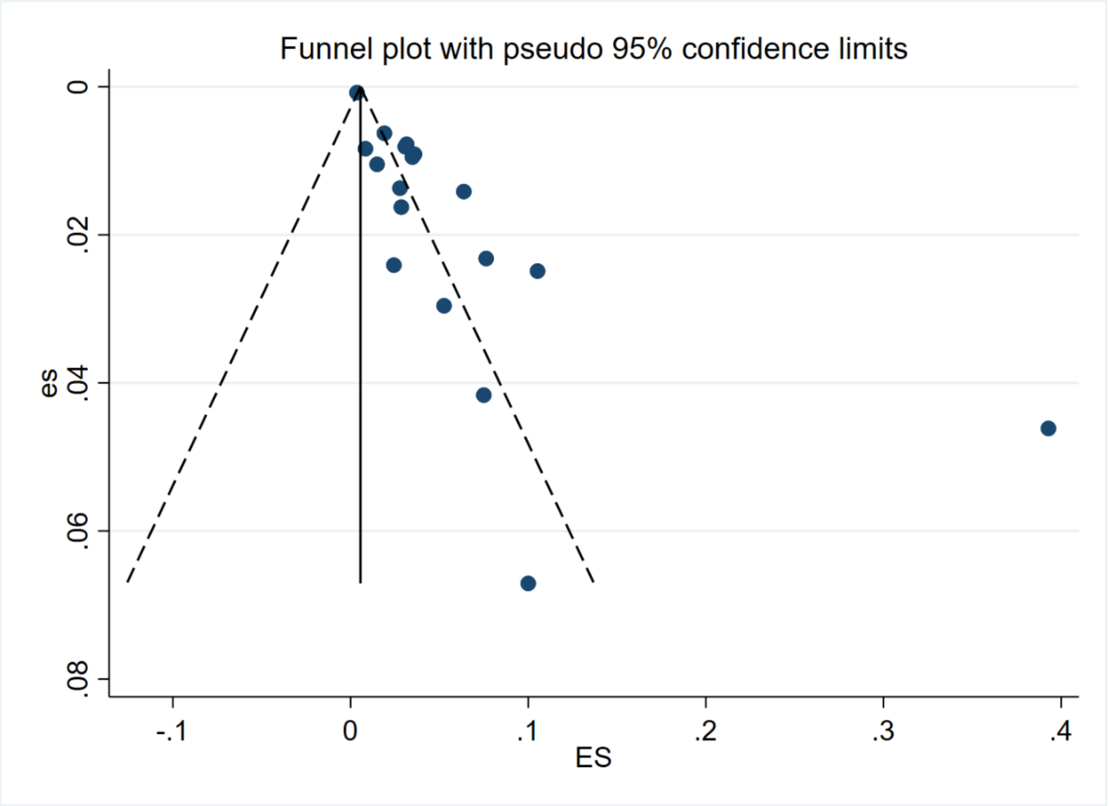

Supplementary figure 4: Funnel plot of the incidence of suspected HIT in ECMO patients

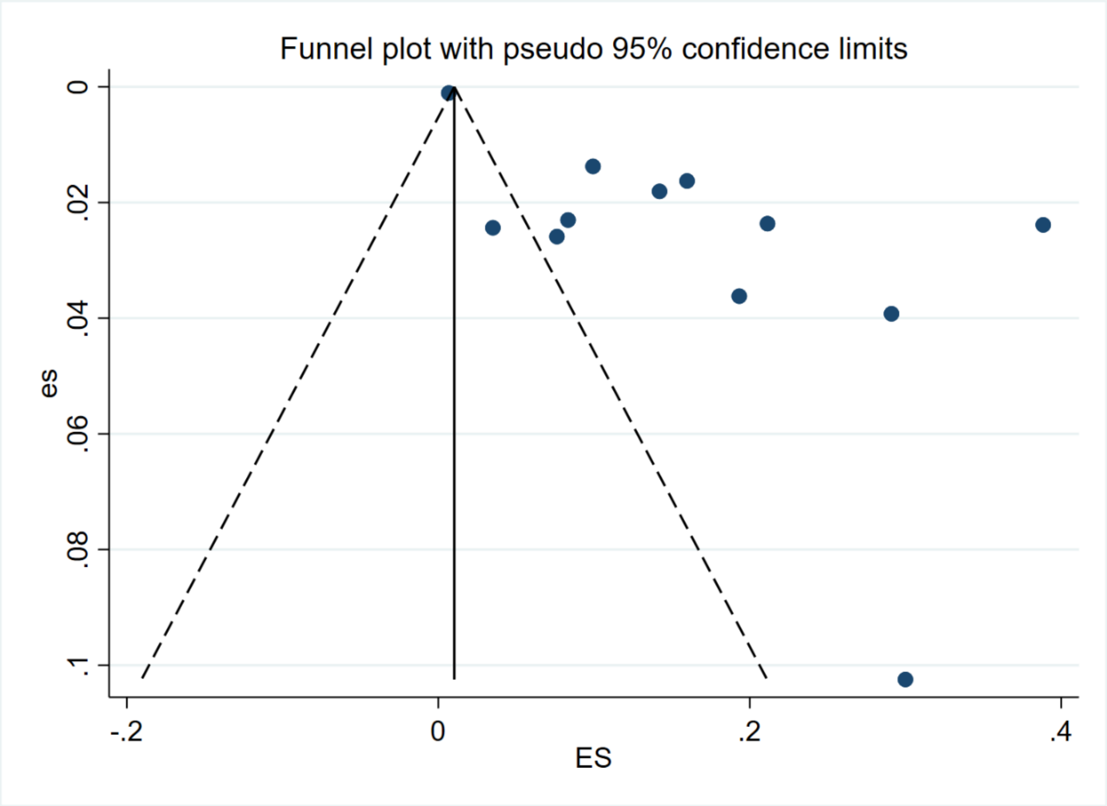

Supplementary figure 5: Funnel plot of the incidence of thrombosis, bleeding and mortality of HIT patients with ECMO-supported.

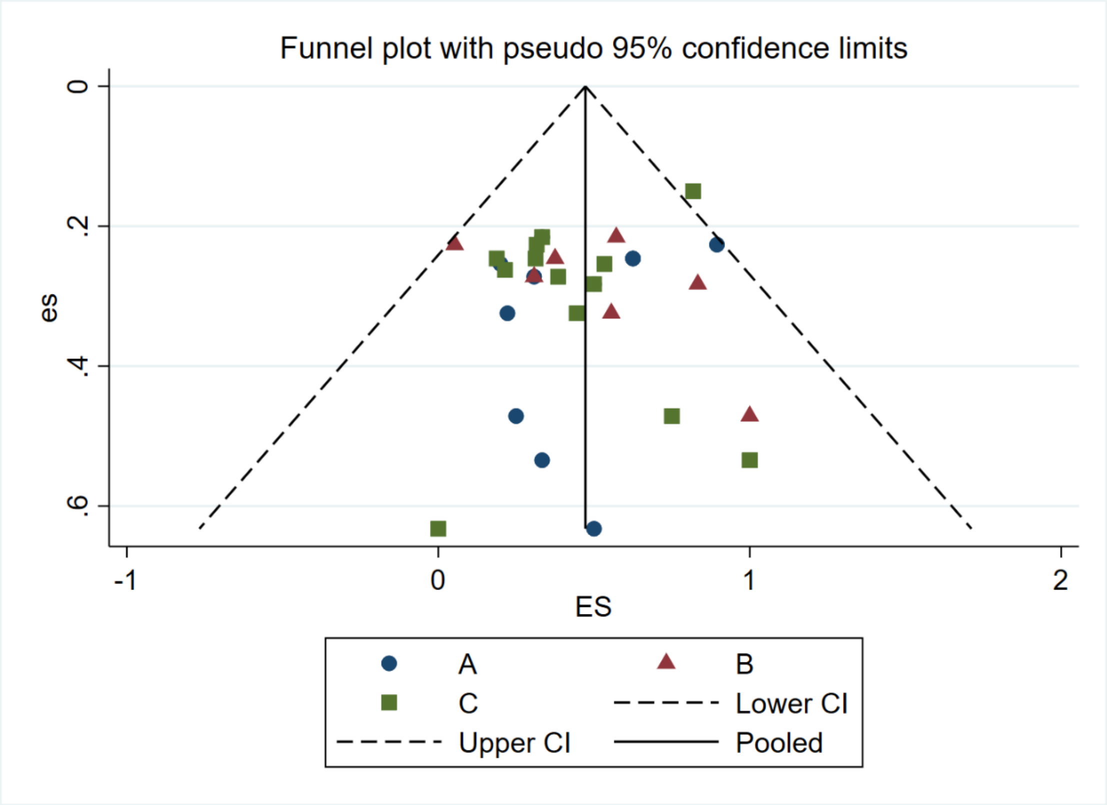

Supplementary figure 6: Funnel plot of the odds ratio of thrombosis, bleeding and death of HIT

patients with ECMO-supported.

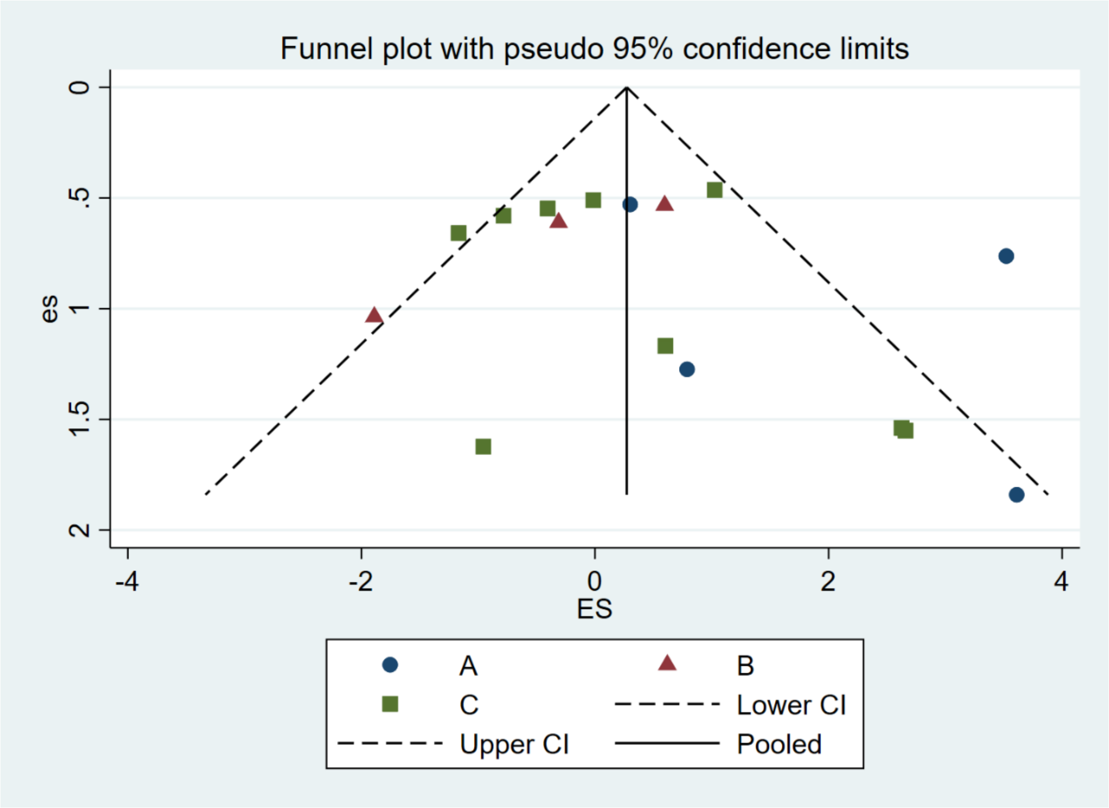

Supplement: Supplementary file 1 — Supplementary Material 1 [file 12959_2024_624_MOESM1_ESM.pdf]
